# 

**Published:** 1881-12

**Authors:** 


CONTENTS.

To Our Readers ... 439
Dangers from Decaying Teeth ... 440
In Sunset Light ... 441
Taking Cold ... 442
Eating, Drinking and Exercise ... 444
Remarkable Operation ... 444
How to Sleep Well ... 445
Sleeplessness and Insanity ... 446
Tinkering the Postal Laws ... 447
The Toys (poetry) ... 447
Samuel Bowles and Dr. Holland ... 448
About Gondolas ... 448
Lafayette at Yorktown ... 449
A Curious Fact ... 449
Facts about Gold ... 450
To Prevent Hair Turning Gray ... 450
An Antidote to Snake Poison ... 451
The Garden and the Farm ... 452
Consumption ... 453
Latent Insanity ... 471
Healthfulness of Fruit ... 473
True Teachings ... 474
Popular Fallacies ... 474
A Sign of Consumption ... 480
Interesting Facts ... 481
Nearsighted ... 483
Influence of Christianity on Medical Science ... 484
A Shave ... 485
Habitual Mouth Breathing ... 486
On Training ... 486
Garfield's Religious Views ... 487
Reconciliation (poetry) ... 487
Wearing the Beard ... 488
Poison of Serpents ... 488
Kindness to Children ... 489
Constipation ... 491

